# Single-cell profiling of blood and cerebrospinal fluid in tuberculous meningitis

**DOI:** 10.1093/jimmun/vkaf186

**Published:** 2025-08-08

**Authors:** Trinh Thi Bich Tram, Lucy C Garner, Le Nguyen Hong Thai, Le Thanh Hoang Nhat, Do Dang Anh Thu, Ho Dang Trung Nghia, Le Hong Van, Guy E Thwaites, Vu Thi Ngoc Ha, Paul Klenerman, Nguyen Thuy Thuong Thuong

**Affiliations:** Oxford University Clinical Research Unit, Ho Chi Minh City, Vietnam; Translational Gastroenterology and Liver Unit, Nuffield Department of Medicine, University of Oxford, Oxford, United Kingdom; Oxford University Clinical Research Unit, Ho Chi Minh City, Vietnam; Oxford University Clinical Research Unit, Ho Chi Minh City, Vietnam; Oxford University Clinical Research Unit, Ho Chi Minh City, Vietnam; Hospital for Tropical Diseases, Ho Chi Minh City, Vietnam; Oxford University Clinical Research Unit, Ho Chi Minh City, Vietnam; Oxford University Clinical Research Unit, Ho Chi Minh City, Vietnam; Centre for Tropical Medicine and Global Health, Nuffield Department of Medicine, University of Oxford, Oxford, UK; Oxford University Clinical Research Unit, Ho Chi Minh City, Vietnam; Translational Gastroenterology and Liver Unit, Nuffield Department of Medicine, University of Oxford, Oxford, United Kingdom; Peter Medawar Building for Pathogen Research, University of Oxford, Oxford, United Kingdom; National Institute for Health and Care Research Oxford Biomedical Research Centre, John Radcliffe Hospital, Oxford, United Kingdom; Oxford University Clinical Research Unit, Ho Chi Minh City, Vietnam; Centre for Tropical Medicine and Global Health, Nuffield Department of Medicine, University of Oxford, Oxford, UK

**Keywords:** CSF, PBMCs, single-cell, scRNA-seq, tuberculosis meningitis

## Abstract

Tuberculous meningitis (TBM) is the most severe form of tuberculosis, with a fatality rate of 20% to 50% in treated individuals. Although corticosteroid therapy can increase survival in HIV-negative people with TBM, better antimicrobial and host-directed therapies are required to improve outcome. There is, therefore, a need to better understand local immunopathologic pathways. Despite its power in identifying disease-specific cellular profiles, single-cell RNA sequencing (scRNA-seq) has been underutilized in cerebral samples in brain infection. We employed scRNA-seq to analyze fresh pretreatment cerebrospinal fluid (CSF) from 4 TBM patients, along with paired PBMCs. While 29 cell subtypes were present in both tissues, their relative abundance varied significantly. In particular, CSF was enriched with highly inflammatory microglia-like macrophages, GZMK^+^CD8^+^ effector-memory T (TEM) cells, and CD56^bright^ NK cells. The latter 2 subsets exhibited reduced cytotoxicity compared with their blood-enriched counterparts, namely cytotoxic GNLY^+^CD8^+^ TEM and CD56^dim^ NK cells, respectively. Across multiple cell types, inflammatory signaling pathways were increased and oxidative phosphorylation was decreased in CSF compared to PBMCs. This study highlights the value of scRNA-seq for exploring CSF immunopathogenesis in TBM patients and offers a resource for future studies investigating the pathophysiology of TBM and other brain infections, including potentially targetable cell populations linked with immune-mediated pathology.

## Introduction

Failures in the host response following inhalation of *Mycobacterium tuberculosis* (*Mtb*) can result in bacterial replication and hematogenous dissemination of bacteria to organs beyond the lung. Tuberculous meningitis (TBM) is one such example, accounting for approximately 5% to 10% of extrapulmonary tuberculosis (TB) cases and 1% of active TB cases.^1^^,^^2^ TBM is the most severe and fatal form of TB, with a mortality rate of 20% to 50% in treated individuals and up to 50% long-term neurological disability among survivors.^3^^,^^4^

Given the lack of direct access to brain tissue in people with TBM, cerebrospinal fluid (CSF) has been used as a proxy to study cellular and immunological events in the brain. CSF can be taken safely and repeatedly by lumbar puncture of the subarachnoid space. Immune responses in TBM were thought to be compartmentalized within the central nervous system (CNS); however, recent transcriptomic studies revealed significant levels of systemic inflammation.^5–7^ Whole blood RNA sequencing (RNA-seq) of TBM patients with HIV coinfection showed increased neutrophil-associated transcripts and inflammasome signaling in those with immune reconstitution inflammatory syndrome compared with those without.^5^ T- and B-cell activation pathways were decreased in the blood of TBM patients with fatal outcomes, but their activities in CSF remain unknown.^6^ RNA-seq of lumbar CSF from children with TBM showed enhanced protein translation and cytokine signaling compared with other CNS infections, while blood of children with TBM exhibited upregulated inflammasome signaling compared with healthy donors.^7^ Given the localized CNS response in TBM, alongside known systemic effects, linking host immune responses in PBMCs and CSF is essential for understanding TBM pathogenesis.

While bulk RNA-seq provides an average gene expression profile of a mixed population of cells, single-cell RNA-seq (scRNA-seq) measures gene expression at the level of individual cells, enabling hypothesis-free identification and characterization of cell type–specific immune responses in human disease, including TB.^8^ In a previous scRNA-seq study, a cytotoxic NK cell subset (*CD7*^+^*GZMB*^+^) was depleted in PBMCs from latent TB compared with healthy individuals, and further depleted in active TB, suggesting a novel biomarker for distinguishing these manifestations.^9^ scRNA-seq of PBMCs from people with pulmonary TB also reveals shifts in immune cell composition with disease severity.^10^ In particular, patients with severe disease showed a marked reduction of Th1 and NK cell subsets compared to milder cases and healthy donors. Despite this reduction, Th1, CD8^+^ T, and NK cells showed signs of dysregulation, with the latter 2 subsets displaying a highly cytotoxic transcriptional phenotype.^10^ Therefore, scRNA-seq offers a promising approach to characterize CSF immunopathogenesis in people with TBM and could reveal novel cell or gene targets for TBM diagnostics and treatment.

Compared with blood, studying the CSF has several challenges, limiting its broad implementation in CNS infection research. Sampling is invasive, and samples are prone to blood contamination and typically contain low numbers of cells.^7^^,^^11^ Consequently, scRNA-seq on CSF in inflammatory diseases is typically performed with fresh samples.^12–16^ A previous study on 2-month-frozen CSF samples showed the potential of using cryopreserved CSF in scRNA-seq, but further validation is required due to significant variation in cell viability and the number of cells recovered.^17^ Additionally, while comprehensive reference datasets of the blood transcriptional landscape in health and numerous diseases are publicly available, a reference dataset for CSF in adult TB is lacking. Herein we evaluated the utility of scRNA-seq to investigate the pathogenesis of TBM by profiling fresh paired PBMCs and CSF from 4 adult TBM patients, alongside PBMCs from 3 healthy controls. We identified 15 major cell types and 29 cell subtypes, each with distinct tissue distribution patterns. We uncovered compartment-specific transcriptional differences between PBMCs and CSF, with multiple CSF cell populations exhibiting enhanced IFN and inflammatory responses and reduced oxidative phosphorylation. Preliminary analysis further revealed increased inflammatory and apoptotic responses, as well as altered metabolism, in specific cell subsets potentially associated with mortality. Together, these findings highlight the value of scRNA-seq for providing mechanistic insights into TBM pathogenesis.

## Materials and methods

### Participants

Of the 4 patients with suspected TBM, 3 were participants of the LAST ACT trial (NCT03100786), which evaluated the impact of adjunctive dexamethasone on outcomes for HIV-negative adults with TBM. This trial was conducted in the Hospital for Tropical Diseases (HTD) and Pham Ngoc Thach Hospital (PNT) in Ho Chi Minh City, Vietnam, from February 2018 to March 2024.^18^ The remaining patient was a participant of an ongoing cross-sectional study, started in May 2023 in the HTD, aimed at improving the diagnosis of CNS infectious diseases. Adults (≥18 years) who underwent lumbar puncture as part of routine care for suspected CNS infection were recruited. Patients were suspected of TBM if they had ≥5 d of meningitis symptoms and abnormal CSF parameters (including color, opening pressure, white blood cell count, protein, lactate, and glucose). The degree of diagnostic certainty was further stratified using the Marais criteria, which incorporate clinical, CSF, radiological, and microbiological findings.^19^ Detailed clinical descriptions of each patient are included in File S1. Three healthy donors were enrolled in an ongoing epidemiology study of human resistance to *Mtb* infection, conducted in the HTD in July 2022. These donors had no historical TB nor any symptoms or signs suggestive of TB disease at the time of enrollment. Written informed consent was obtained from all participants or their relatives if they were incapacitated. Study protocols were approved by the HTD and PNT hospital in Vietnam, and the Oxford Tropical Research Ethics Committee, United Kingdom.

### Sample collection

As part of routine hospital diagnostic procedures, 5 to 10 mL of CSF and 1 mL heparinized blood was collected before the start of antimicrobial and anti-inflammatory treatment. Two to 3 mL of CSF was sent to the hospital laboratory for routine tests for viral, bacterial, or fungal infection. The remaining CSF was concentrated by centrifugation at 300 × g for 10 min and resuspended in 700 µL of CSF supernatant. This CSF deposit was used for TB diagnostic tests, including 100 μL for Ziehl-Neelsen smear and 200 μL for Xpert MTB/RIF Ultra, and 200 μL was stored at −80 °C for future use. The remaining 200 μL of CSF was used for scRNA-seq if processed within 4 hours of collection, with ≥75 cells/µL deposit, and no artificial blood contamination (evidenced by >200 RBCs/μL CSF total).^15^ After recentrifugation (300 × *g*, 5 min), CSF pellets were resuspended in 50 μL PBS containing 0.04% BSA (Sigma) and counted. Blood was processed alongside CSF, with PBMCs isolated using density gradient centrifugation with Histopaque 1077 (Sigma) following the manufacturer’s instructions. Both PBMCs and CSF were used fresh for downstream applications, including scRNA-seq and flow cytometry.

### Flow cytometry

PBMCs and CSF cells were stained with Zombie Aqua dye (BioLegend), then incubated with a cocktail of antibodies for 15 min at room temperature: CD3-PerCP (SK7), CD4-FITC (RPA-T4), CD8-APC/Cy7 (SK1), CD14-BV605 (63D3), CD16-PE/Cy7 (3G8), CD19-Alexa Fluor 700 (SJ25C1), CD56-BV421 (HCD56), CD123-PE (6H6), and CD11c-APC (SHCL-3). CD3-PerCP, CD4-FITC, and CD11c-APC were from BD Biosciences, while all other antibodies and dyes were from BioLegend. Samples were run on a BD FACSLyric flow cytometer using FACSuite acquisition software. Data were analyzed using FlowJo software (v10.9.0, BD) using the gating strategy depicted in Fig. S1.

### Generation and sequencing of 10x Genomics scRNA-seq libraries

Single-cell suspensions were loaded onto a 10x Genomics Chromium Controller. One sample was loaded per channel with a target capture of 4,000 cells, except for PBMCs from one healthy control that had a target capture of 1,000 cells. Libraries were generated using the Chromium Next GEM Single Cell 3′ Reagent Kit v3.1 according to the manufacturer’s instructions. The library from the low-capture healthy control PBMC sample was sequenced internally on an Illumina MiSeq to a mean depth of 20,922 reads per cell, with a sequencing saturation of 66.1%. All other libraries were sequenced on an Illumina NovaSeq 6000 (BGI, Hong Kong) to a mean depth of 33,072 to 163,448 reads per cell, with a sequencing saturation of 54.0% to 91.6%.

### scRNA-seq analysis

#### Preprocessing and quality control

Sequencing data were processed using Cell Ranger v7.1.0 (10x Genomics). Namely, reads were aligned to a GRCh38 reference genome (v3.0.0) and gene expression matrices generated using *cellranger count*. SoupX (v1.6.2) was used to remove ambient RNA contamination.^20^ Doublets were removed using Scrublet (v0.0.4) with default parameters.^21^ The data from all samples were combined using the merge function in Seurat v4.3.1.^22^ Low-quality cells with <500 genes detected or >10% of reads aligned to the mitochondrial genome were removed. Genes expressed in <5 cells were removed.

#### Data normalization, dimensionality reduction, and clustering

After quality control, data were analyzed using Seurat v4.3.1.^22^ Raw count data were normalized using sctransform^23^ with percent mitochondrial reads and the number of unique molecular identifier counts regressed out. Variance stabilizing transformation was performed to identify the 3,000 highly variable genes, which were used for principal component analysis. Batch effects from different donors were removed using Harmony.^24^ A shared nearest-neighbor graph was constructed using the top 30 Harmony components, and clustering was performed with the Louvain algorithm (resolution 1.9). Data were visualized using uniform manifold approximation and projection (UMAP) of the top 30 Harmony principal components.

For some analyses, we combined the data from TBM patients with that from 3 healthy donors. Before merging, raw counts from the healthy donors were subjected to the same quality control metrics as the TBM dataset. The combined dataset was processed using the same procedures as for the TBM dataset, with the exception that 4,000 highly variable genes were used for UMAP visualization.

#### Cell annotations

Clusters were annotated at 2 levels: broad annotation of major cell types and deeper annotation of cell subtypes. For the first level, data were mapped to reference datasets of PBMCs and immune cells (Monaco and Blueprint/ENCODE) using Azimuth^22^ and SingleR^25^ algorithms, respectively. To improve annotation accuracy, we manually inspected the expression of known cell type marker genes. Additionally, we identified and reviewed the genes differentially expressed between clusters (*FindAllMarkers* function with MAST^26^). The MAST model included patient identifier and cellular detection rate (CDR) as covariates. Genes with a fold change ≥1.25 and a false discovery rate (FDR) <0.05 were considered significantly enriched in a cluster.

In the combined dataset of cells from TBM patients and 3 healthy donors, cell types for the healthy donors were inferred by mapping to a reference of PBMCs from the 4 TBM patients using SingleR.

#### Differential abundance analysis

To formally compare the cell type proportions among PBMCs and CSF, differential abundance testing was performed using miloR (v1.9.1)^27^ with parameters k = 30, d = 30, and prop = 0.1. The design was ∼ tissue. The *plotDAbeeswarm* function was used with mixed populations removed. Neighborhoods with a spatial FDR <0.1 were considered significantly differentially abundant.

#### Differential expression analysis

Differentially expressed genes (DEGs) between PBMCs and CSF for each cell type, as well as DEGs between cell subtypes, were identified using the *FindMarkers* function from Seurat with MAST,^26^ including patient identifier and CDR as covariates. Cell types with <10 cells were excluded from the analysis. Only genes detected in >1% of cells in each cell type in a given tissue were included. Ribosomal and mitochondrial genes were excluded from the analysis. Significant genes were defined as those with a fold change ≥1.25 and an FDR <0.05.

#### Gene set enrichment analysis and overrepresentation analysis

Gene set enrichment analysis was conducted using clusterProfiler (v4.6.2)^28^ with the fast gene set enrichment analysis (FGSEA) method.^29^ For each cell type, all genes were ranked in descending order by their log_2_ fold change values. “Hallmark” gene sets were obtained from the Molecular Signatures Database (MSigDB) (http://www.gsea-msigdb.org/gsea/index.jsp). Pathways with an FDR <0.05 were considered significant. Overrepresentation analysis (ORA) for Gene Ontology terms was conducted using clusterProfiler (v4.6.2) with DEGs as input. The background gene list comprised genes expressed in at least 5 cells in the dataset.

## Results

### Patient characteristics

**Figure 1.**
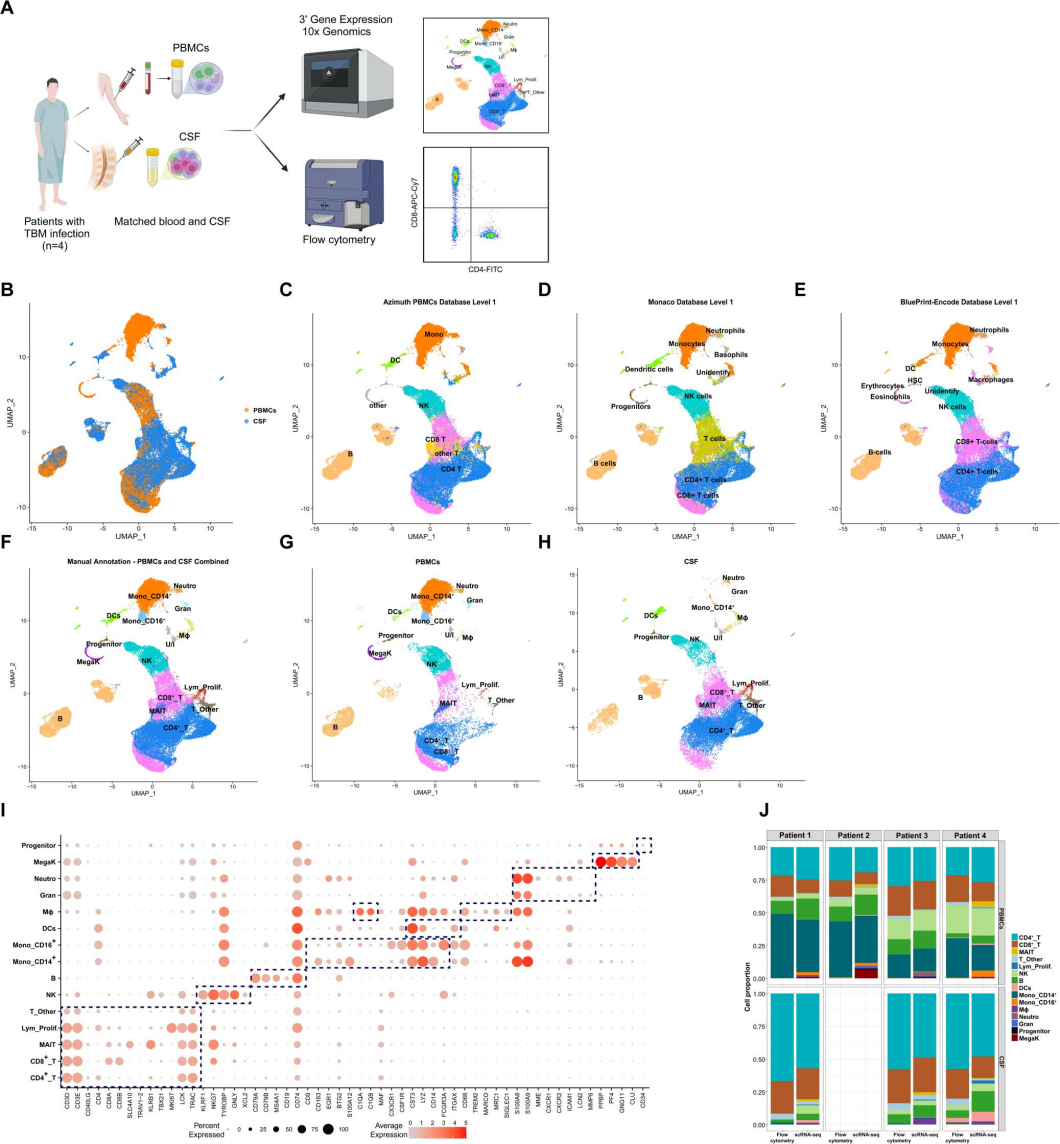
Main cell types (level 1) in combined PBMCs and CSF, and comparison with flow cytometry results. (A) Experimental workflow. Figure generated using BioRender. (B) UMAP plot of all cells colored by tissue of origin. (C–E) UMAP showing level 1 annotation of cell types for combined PBMCs and CSF based on reference datasets: (C) Azimuth for PBMCs, (D) Monaco for immune cells, and (E) Blueprint/ENCODE for stromal and immune cells. (F–H) UMAP showing level 1 annotation of cell types based on expert knowledge for (F) combined PBMCs and CSF, or separated into (G) PBMCs and (H) CSF. (I) Dot plot depicting selected marker genes of major cell types (indicated by dashed boxes). Dot color indicates the mean normalized expression and dot size indicates the fraction of cells expressing the gene. (J) Comparison of cell type proportions identified by flow cytometry and scRNA-seq. CSF flow cytometry data are not available for patient 2. B, B cells; CD4^+^_T, CD4^+^ T cells; CD8^+^_T, CD8^+^ T cells; DCs, dendritic cells; Gran, mixed granulocytes; Lym_Prolif., proliferative lymphocytes; MAIT, MAIT cells; MegaK, megakaryocytes; Mono_CD14^+^, CD14^+^ monocytes; Mono_CD16^+^, CD16^+^ monocytes; MΦ, macrophages; Neutro, neutrophils; NK, NK cells; T_Other, other T cells; U/I, unidentified.

To analyze the cell composition and transcriptional landscape of PBMCs and CSF in TBM, we performed scRNA-seq on fresh paired pretreatment samples from 4 patients (Fig. 1A). Baseline characteristics of the patients are summarized in Table 1. All 4 patients were clinically diagnosed with TBM, with microbiological confirmation in 2 cases. White blood cells ranged from 7 × 10^6^ to 11 × 10^6^ cells/mL of blood, with neutrophils comprising the largest proportion (∼80%), followed by lymphocytes. All 4 patients exhibited a high white cell count in CSF (∼300 × 10^3^ cells/mL), with lymphocytes being the predominant cell type (>80%). Three patients survived, while one died within a week of hospitalization.

**Table 1.** Baseline characteristics of 4 patients with tuberculous meningitis.

Characteristic	TBM patients (n = 4)				Healthy donors (n = 3)		
	Patient 1	Patient 2	Patient 3	Patient 4	Donor 1	Donor 2	Donor 3
**Age (years)**	40	60	40	51	26	34	27
**Sex**	Male	Male	Male	Male	Male	Male	Male
**HIV infection**	Negative	Negative	Negative	Negative	Negative	Negative	Negative
**CSF diagnostics**							
** Ziehl-Neelsen stain**	Positive	Negative	Negative	Negative	–	–	–
** Xpert MTB/RIF Ultra**	Trace Ct1 = 25.5	Low Ct1 = 22.8 Ct2 = 25.4	Negative	Negative	–	–	–
** MGIT culture**	Negative	Positive	Negative	Not done	–	–	–
** Cryptococcal antigen LFA**	Negative	Negative	Negative	Negative	–	–	–
**CSF cell counts**							
** Leukocytes (10^3^ cells/mL)**	382.0	140.0	330.0	357.0	–	–	–
** Neutrophils (%)**	13.0	0.0	0.0	8.0	–	–	–
** Lymphocytes (%)**	87.0	100	100	92.0	–	–	–
** Eosinophils (%)**	0.0	0.0	0.0	0.0	–	–	–
**Blood cell counts**							
** Leukocytes (10^6^ cells/mL)**	11.4	7.5	11.3	7.8	6.62	4.52	6.67
** Monocytes (%)**	4.0	5.4	4.9	8.2	6.3	6.2	5.1
** Neutrophils (%)**	79.9	84.4	81.7	56.4	48.7	49.5	56.4
** Lymphocytes (%)**	12.0	10.0	13.0	27.4	40.5	41.2	31.8
** Eosinophils (%)**	2.0	0.1	0.3	5.2	3.9	2.4	6.9
**Diagnostic category^a^**	Definite TBM	Definite TBM	Probable TBM	Possible TBM	–	–	–
**MRC grade^b^**	Grade 2	Grade 2	Grade 1	Grade 2	–	–	–
**Treatment outcome at the end of anti-TB therapy**	Survived	Died within 1 wk of hospitalization	Survived	Survived	–	–	–

TBM patients received standard anti-TB therapy along with adjunctive dexamethasone treatment, in accordance with World Health Organization guidelines.

Ct1: Ct value of *IS6110*/*IS1081* for *Mtb* detection.

Ct2: Ct value of earliest *rpoB* probes, used as a semi-quantitative estimate of *Mtb* burden in the sample.^30^ No *rpoB* signal was detected in CSF from patient 1.

LFA, lateral flow assay; MGIT, Mycobacteria Growth Indicator Tube.

a Diagnostic categories were assigned according to the consensus case definition.^19^

b MRC grade denotes modified British Medical Research Council criteria.

### Broad annotation (level 1) of immune cell types in blood and CSF

A total of 19,921 and 21,292 single-cell transcriptomes were obtained from matched PBMCs and CSF, respectively. The number of genes detected per cell was comparable between PBMCs and CSF (2,723 ± 226 and 2,773 ± 550, respectively) (Table S1). Data were of a high quality, with low percentages of doublets (<5%), ambient RNA (2%), and cells with >10% mitochondrial reads (<2%) (Fig. S2). After removal of low-quality cells, 17,264 PBMCs and 20,100 CSF cells were combined for analysis (Fig. 1B).

PBMCs and CSF cells partially overlapped on the UMAP (Fig. 1B), suggesting a mixture of shared and compartment-specific cell types. As there is no reference CSF database for cell annotation, we reference mapped combined PBMCs and CSF to the Azimuth PBMCs reference using Azimuth (Fig. 1C), and to Monaco and Blueprint/ENCODE references using SingleR (Fig. 1D, E). The number of detected cell types varied with each reference: Azimuth annotated 7 major cell types while the others annotated 11. Common clusters included B cells, CD4^+^ T cells, CD8^+^ T cells, monocytes, and NK cells. However, inconsistencies in annotations were observed. For instance, Monaco identified progenitors, whereas Blueprint/ENCODE labeled the same cells as eosinophils and erythrocytes, and Azimuth did not recognize them. Additionally, due to the lack of neutrophils in PBMCs, Azimuth was unable to annotate neutrophils. To reconcile annotation differences, we further manually annotated cell types using expert knowledge (Table S2). Louvain clustering at resolution 1.9 produced 41 clusters (Fig. S3), which were subsequently classified into 15 major cell types based on canonical marker gene expression (Fig. 1F–I). Identified cell types included B cells (*CD79A*/*B*, *MS4A1*, *CD74*, *CD19*), CD4^+^ T cells (*CD3D*/*E*, *CD4*), CD8^+^ T cells (*CD3D*/*E*, *CD8A*/*B*), mucosal-associated invariant T (MAIT) cells (*SLC4A10*), proliferative lymphocytes (*MKI67*), “T_Other” cells (possibly dead cells due to low RNA content, Fig. S4), NK cells (*KLRF1*, *GNLY*, *XLC2*), dendritic cells (DCs) (*CST3*, *LYZ*, *ITGAX*), 2 types of monocytes (CD14^+^ [*CD14*] and CD16^+^ [*FCGR3A*]), macrophages (*C1QA*, *C1QB*, *MARCO*, *SIGLEC1*, *TREM2*, *MRC1*), megakaryocytes (*PPBP*, *PF4*, *GNG11*, *CLU*), progenitors (*CD34*), and 2 granulocyte types (neutrophils: *S100A8*, *S100A9*, *ICAM1*, *CXCR2*, *MME*; mixed granulocytes: *MMP8*, *LCN2*). All cell types were present in both PBMCs and CSF, except for megakaryocytes and CD16^+^ monocytes, which were only found in blood (Fig. 1G, H).

We validated the cell composition identified using scRNA-seq with flow cytometry (Fig. 1J). Flow cytometry detected CD4^+^ T cells, CD8^+^ T cells, and other CD3^+^ T cells. As anticipated, scRNA-seq provided a higher resolution of cell subtypes compared with flow cytometry, but there was high concordance in the frequencies of shared cell types. Overall, microfluidics-based scRNA-seq successfully characterized the cell composition of PBMCs and CSF from adult TBM patients.

### High-resolution annotation (level 2) of immune cell subtypes in blood and CSF

**Figure 2.**
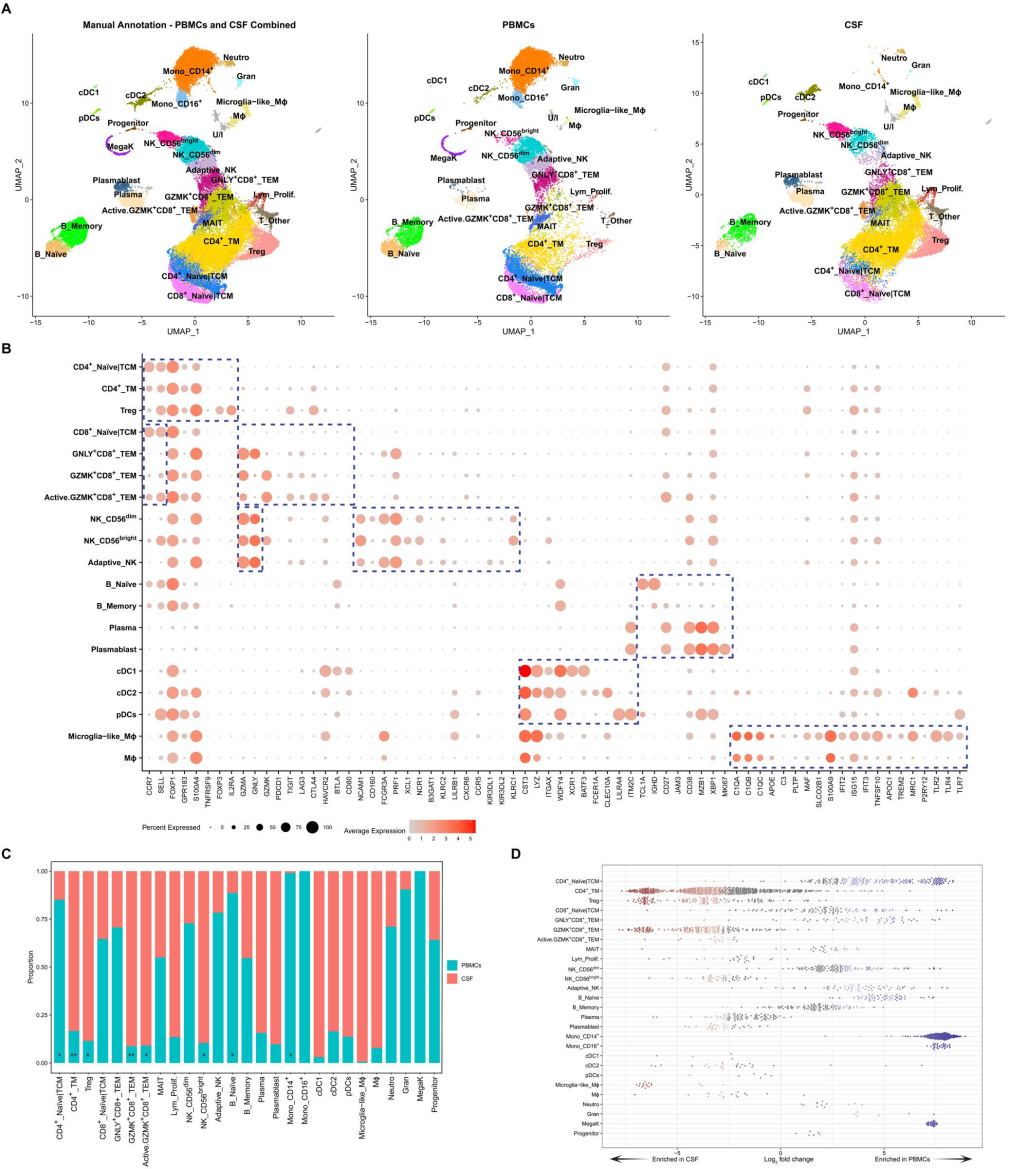
Cell subtypes (level 2) in combined PBMCs and CSF. (A) UMAP plot of high-resolution annotation (level 2) of cell subtypes from the main cell types, based on expert knowledge for combined PBMCs and CSF cells (left), PBMCs (middle), or CSF cells (right). (B) Dot plot depicting selected signature genes of cell subtypes (indicated by dashed boxes). Dot color indicates the mean normalized expression and dot size indicates the fraction of cells expressing the gene. (C) The proportions of cell subtypes in PBMCs and CSF cells from all 4 TBM patients. Comparison of cell subtype proportions between PBMCs and CSF was performed using paired *t*-tests. *P < 0.05, **P < 0.01. (D) Relative abundance of neighborhoods identified using the Milo k-NN weighted-network algorithm. Neighborhoods enriched in CSF are in red, those enriched in PBMCs are in blue, and those with no significant enrichment are in gray. Adaptive_NK, adaptive-like NK cells; cDC1, conventional DC1; cDC2, conventional DC2; Gran, mixed granulocytes; Lym_Prolif., proliferative lymphocytes; MegaK, megakaryocytes; MΦ, macrophages; Neutro, neutrophils; pDCs, plasmacytoid DCs; TCM, T central memory cells; TEM, T effector memory cells; TM, T memory cells; U/I, unidentified.

At the broad level of annotation, multiple Louvain clusters were identified within B cells, NK cells, CD4^+^ T cells, CD8^+^ T cells, and DCs, suggesting the presence of cell subtypes (Fig. 1F, Fig. S3). We further annotated the subtypes based on marker gene expression (Fig. 2A, B). This considerably increased cell annotation granularity to 29 subtypes (Fig. 2A). For example, we identified 4 subsets of B cells: naïve (*TCL1A*, *IGHD*), memory (*JAM3*), plasmablast (*CD27*, *CD38*, *XBP1*, *MZB1*, *MKI67*), and plasma cells (similar to plasmablast, but negative for *MKI67*). CD4^+^ T cells divided into 3 subtypes: CD4^+^ naïve/T central memory (TCM) (*CCR7*, *SELL*), T regulatory (Treg) (*FOXP3*, *IL2RA*), and CD4^+^ memory (CD4^+^ TM) (*GPR183*, *S100A4*). CD8^+^ T cells comprised 4 clusters: CD8^+^ naïve/TCM (*CCR7*, *SELL*) and 3 effector-memory populations, ie GNLY^+^CD8^+^ effector-memory T (TEM) (high *GNLY*), GZMK^+^CD8^+^ TEM (high *GZMK*, low *GNLY*), and activated GZMK^+^CD8^+^ TEM (high inhibitory receptors: *TIGIT*, *LAG3*, *CTLA4*, *HAVCR2*). DCs included 3 subtypes: conventional DC1 (cDC1; *ITGAX*, *WDFY4*, *XCR1*, *BATF3*), conventional DC2 (cDC2; *ITGAX*, *FCER1A*, *CLEC10A*), and plasmacytoid DCs (pDCs; *LILRA4*, *ITM2C*). Three subtypes of NK cells were identified: immunomodulatory CD56^bright^ NK (high *NCAM1*, *CD160*, *XCL1*), cytotoxic CD56^dim^ NK (moderate *NCAM1*, *CD160*), and adaptive-like NK^31^^,^^32^ (moderate *NCAM1*, *B3GAT1*, *LILRB1*, very low *KLRC1*). Macrophages were divided into 2 clusters: macrophages (low *C1QA*/*B*/*C*) and microglia-like macrophages (high *C1QA*/*B*/*C*, *APOE*, *MRC1*, *TLR2*, *TLR4*). Except for monocytes and rare cell types like cDC2, most subtypes were present in PBMCs and CSF in all 4 patients (Fig. S5). Overall, CSF comprised a large variety of cell subtypes, with comparable diversity to blood.

### Relative abundance of cell types in blood and CSF

We next analyzed differences in cell composition between PBMCs and CSF, focusing on the 27 cell subtypes annotated at high resolution. The unidentified group and “T_Other” (Fig. S4) were excluded from the analysis. As expected, megakaryocytes were absent in CSF. CD14^+^ monocytes and CD16^+^ monocytes were almost exclusive to PBMCs, while microglia-like macrophages were mainly in CSF (Fig. 2C). The following cell clusters predominantly comprised cells from CSF in all patients: Treg (*P* = 0.01), CD4^+^ TM (*P* = 0.008), GZMK^+^CD8^+^ TEM (*P* = 0.009), activated GZMK^+^CD8^+^ TEM (*P* = 0.003), and NK CD56^bright^ (*P* = 0.03). In contrast, CD4^+^ naïve/TCM (*P* = 0.01) and naïve B (*P* = 0.04) were overrepresented in PBMCs (Fig. 2C, Fig. S6).

Limitations in defining the appropriate cell clustering resolution for annotation can hinder the identification of condition-specific cell subtypes or cell states.^27^ Therefore, we performed formal differential abundance analysis to compare cell proportions in PBMCs and CSF within local neighborhoods using the Milo k-nearest neighbor (k-NN) approach. Among 2,896 neighborhoods spanning the k-NN graph generated from PBMCs and CSF cells, Milo identified 1,845 neighborhoods with differential abundance (spatial FDR <10%) (Fig. S7). Consistent with the annotated cell type–based analysis, neighborhoods of microglia-like macrophages, CD4^+^ TM, and Treg cells were enriched in CSF (Fig. 2D). Conversely, neighborhoods of monocytes (CD14^+^ and CD16^+^), naïve/TCM cells (CD4^+^ and CD8^+^), and naïve B cells were enriched in PBMCs. Notably, GZMK^+^CD8^+^ TEM, activated GZMK^+^CD8^+^ TEM, and CD56^bright^ NK cells were almost exclusive to CSF. In contrast, GNLY^+^CD8^+^ TEM, adaptive-like NK, and CD56^dim^ NK cells were abundant in blood and relatively depleted in CSF. Some neighborhoods of plasma cells, plasmablasts, and subsets of DCs were enriched in CSF. Collectively, these data demonstrate highly compartment-specific cell compositions.

**Figure 3.**
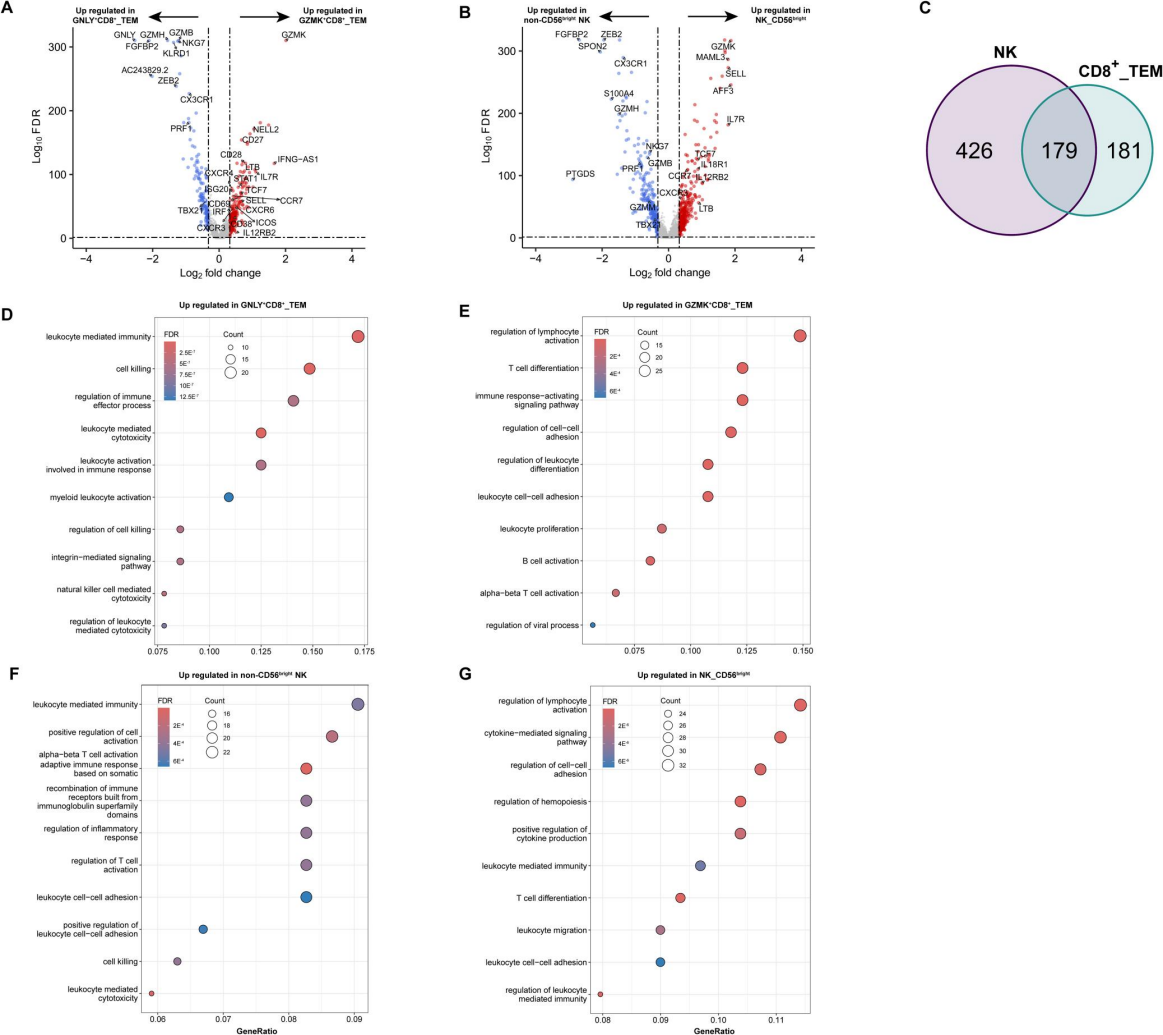
Differential expression of genes and pathways in effector memory CD8^+^ T cells and NK cells. (A, B) Volcano plot showing DEGs between (A) GNLY^+^CD8^+^ TEM and GZMK^+^CD8^+^ TEM cells, and between (B) non-CD56^bright^ NK (including CD56^dim^ and adaptive-like NK cells) and CD56^bright^ NK cells. Top 10 and additional example DEGs are labeled. (C) Number of unique and common DEGs between the 2 effector memory CD8^+^ T-cell populations and the 2 NK cell populations. (D, E) ORA for upregulated genes in (D) GNLY^+^CD8^+^ TEM and (E) GZMK^+^CD8^+^ TEM cells. (F, G) ORA for upregulated genes in (F) non-CD56^bright^ NK and (G) CD56^bright^ NK cells. Top 10 upregulated Gene Ontology Biological Process terms are shown. Gene ratio indicates the fraction of DEGs in the gene set.

We further investigated site-specific differences in effector-memory CD8^+^ T cells and NK cells. A total of 360 genes were differentially expressed between GZMK^+^CD8^+^ TEM and GNLY^+^CD8^+^ TEM cells (Table S5). Among these, the CSF-exclusive GZMK^+^CD8^+^ TEM population significantly upregulated granzyme K (*GZMK*), markers for naïve/TCM cells (*SELL*, *TCF7*, *CCR7*), co-stimulatory receptors (*CD27*, *CD28*, *ICOS*), cytokines (*LTB*), cytokine and chemokine receptors (*IL7R*, *IL12RB2*, *CXCR3*/*4*/*6*), activation markers (*CD38*, *CD69*), and IFN-stimulated genes (*STAT1*, *ISG20*, *IRF1*) (Fig. 3A, Fig. S8). In contrast, genes encoding cytotoxic molecules (*GZMB*/*H*, *GNLY*, *PRF1*), cytotoxicity-related genes (*FGFBP2*, *NKG7*), and markers associated with effector functions and terminal differentiation (*CX3CR1*, *TBX21* [encoding T-bet], *ZEB2*) were upregulated in GNLY^+^CD8^+^ TEM cells (Fig. 3A, Fig. S8). CD56^bright^ NK cells differentially expressed 605 genes compared with non-CD56^bright^ NK cells (including both CD56^dim^ and adaptive-like NK cells) (Table S6), 179 of which overlapped with the DEGs between GZMK^+^CD8^+^ TEM and GNLY^+^CD8^+^ TEM cells (Fig. 3B, C). Similar to GZMK^+^CD8^+^ TEM cells, CD56^bright^ NK cells showed upregulation of *GZMK*, naïve/TCM markers (*SELL*, *TCF7*, *CCR7*), cytokines (*LTB*), and cytokine and chemokine receptors (*IL7R*, *IL12RB2*, *IL18R1*, *CXCR3*) (Fig. 3B, Fig. S8). In contrast, non-CD56^bright^ NK cells, enriched in PBMCs, expressed cytotoxicity-related genes (*GZMB*/*H*/*M*, *PRF1*, *FGFBP2*, *NKG7*) and genes associated with effector differentiation (*CX3CR1, TBX21*, *ZEB2*) (Fig. 3B, Fig. S8), analogous to the GNLY^+^CD8^+^ population. ORA revealed an enrichment of Gene Ontology terms related to T-cell differentiation, cytokine-mediated signaling, and cytokine production in GZMK^+^CD8^+^ TEM and CD56^bright^ NK cells, while cytotoxicity/cell killing pathways were upregulated in GNLY^+^CD8^+^ TEM and non-CD56^bright^ NK cells (Fig. 3D–G).

### Enrichment of activation pathways and altered metabolism in CSF compared with PBMCs

**Figure 4.**
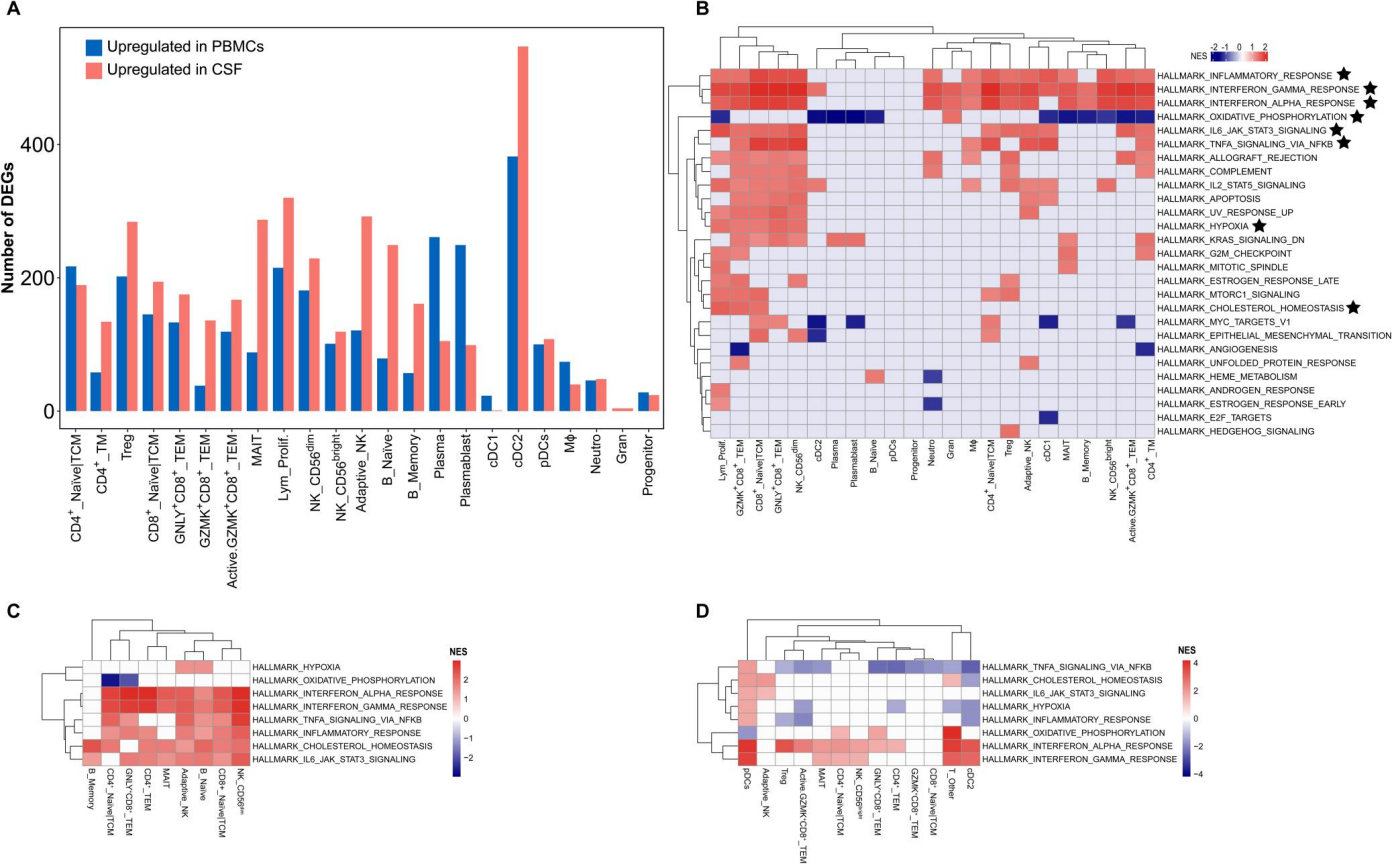
Differential expression of genes and pathways in individual cell subtypes from PBMCs and CSF of TBM patients. (A) Number of upregulated genes in individual cell subtypes in PBMCs and CSF. (B) FGSEA for each cell subtype between PBMCs and CSF from TBM patients. Heatmap shows shared differentially active MSigDB Hallmark gene sets. Red indicates upregulation in CSF, blue indicates upregulation in PBMCs, and gray indicates no significant difference between PBMCs and CSF. Stars indicate gene sets discussed in the text. (C) FGSEA for each cell subtype between CSF from TBM patients and PBMCs from healthy donors. (D) FGSEA for each cell subtype in PBMCs between TBM patients and healthy donors. Color scheme is the same as in (B). Red indicates upregulation in TBM patients, blue indicates upregulation in healthy donors, and gray indicates no significant difference between TBM patients and healthy donors. Gene sets marked by asterisks in (B) are shown in (C) and (D). cDC1, conventional DC1; cDC2, conventional DC2; Gran, mixed granulocytes; Lym_Prolif., proliferative lymphocytes; MegaK, megakaryocytes; MΦ, macrophages; Neutro, neutrophils; pDCs, plasmacytoid DCs; TCM, T central memory cells; TEM, T effector memory cells; TM, T memory cells.

We next determined compartment-specific transcriptional differences at the cell subtype level by identifying DEGs between PBMCs and CSF using MAST.^26^ Since CD16^+^ monocytes and microglia-like macrophages were found exclusively in PBMCs and CSF, respectively (Fig. 2C, D), analysis could not be performed for these clusters. Moreover, due to the significantly lower number of CD14^+^ monocytes in CSF compared with PBMCs (40 vs 4,230), we did not perform differential expression analysis for this cluster. Of the 29,000 genes detected, 1,834 (6.3%) genes were significantly differentially expressed (FDR <0.05, fold change ≥1.25) between PBMCs and CSF (Fig. 4A). In general, more genes were upregulated in CSF compared with PBMCs, suggesting increased cell activity in CSF. The rare cDC2 subset had the largest number of DEGs, with 500 genes upregulated in CSF and 400 genes in PBMCs. Integrated analysis revealed 408 universal DEGs (differentially expressed in ≥5 cell types) (Table S3) and 1,426 cell subtype-specific genes (Table S4). Universal DEGs upregulated in CSF were involved in lymphocyte migration (*CXCR6*) and activation (*CD69*), type I and II IFN responses (*STAT1*, *IRF1*, *SOCS1*, *GBP1*/*2*/*4*/*5* for both; *ISG15*, *ISG20* for type I IFN), neuroinflammation and neurodegeneration (*CD38*),^33^ cytotoxicity (*APOL6*,^34^  *GZMB*), and T-cell activation/exhaustion (*LAG3*) (Table S3). Universal DEGs upregulated in PBMCs were associated with cell proliferation and polarization (*RIPOR2*), migration (*TAGLN2*, *CRIP1*), cell activation and differentiation (*KLF2*, *CD52*), metabolite exchange (*UCP2*), and inflammation and tissue repair (*S100A4*/*10*) (Table S3). These findings reveal compartment-specific transcriptional landscapes in PBMCs and CSF, alongside differences in cell composition.

To further characterize the DEGs, we performed functional enrichment analysis using FGSEA^29^ with MSigDB hallmark gene sets (Fig. 4B). Consistent with the universal DEGs, gene sets related to inflammation and IFN-α/γ (type I/II) responses were enriched in multiple T and NK cell subsets, and in macrophages, in CSF. Likewise, IL-6-JAK-STAT3 and TNF signaling pathways were upregulated in CSF across several T and NK cell subsets, as well as in cDC1s. Intriguingly, hypoxia and cholesterol homeostasis pathways were enriched in several cell types in CSF, while oxidative phosphorylation was consistently downregulated. Similar enrichment patterns were also evident when comparing CSF from TBM patients with PBMCs from healthy donors (Fig. 4C, Fig. S9), highlighting a compartment-specific immune response associated with TBM. Unexpectedly, PBMCs from TBM patients showed reduced inflammatory and TNF signaling, but enhanced IFN-α/γ responses, in several subsets of T and NK cells compared with healthy controls (Fig. 4D).

**Figure 5.**
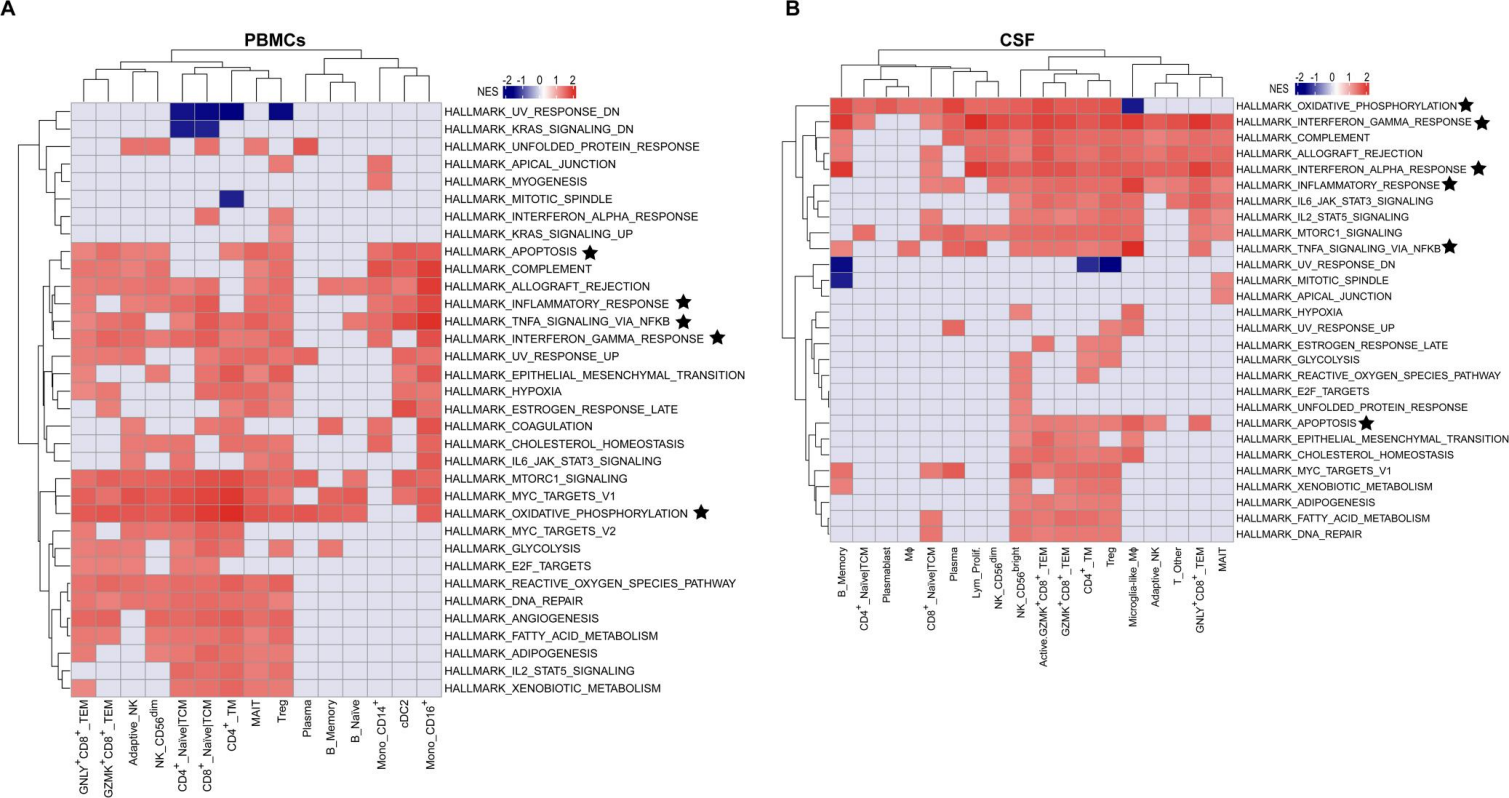
FGSEA in individual cell subtypes from survivors and a nonsurvivor TBM patient. (A, B) FGSEA for each cell subtype in (A) PBMCs and (B) CSF between survivors and a nonsurvivor. Heatmap shows shared differentially active MSigDB Hallmark gene sets. Red indicates upregulation in the nonsurvivor, blue indicates upregulation in survivors, and gray indicates no significant difference between survivors and the nonsurvivor. Stars indicate gene sets discussed in the text. cDC2, conventional DC2; Lym_Prolif., proliferative lymphocytes; MΦ, macrophages; TCM, T central memory cells; TEM, T effector memory cells; TM, T memory cells.

Among the 4 TBM patients, one succumbed to the disease. PBMCs and CSF from the nonsurvivor were enriched (heterogeneously across cell types) for a number of hallmark gene sets associated with the immune response and metabolism. Namely, in PBMCs, activity of inflammatory and IFN-γ and TNF signaling pathways was increased in subsets of T cells, NK cells, and monocytes (Fig. 5A). Enrichment of these pathways, along with the IFN-α response, was also observed across multiple cell types in the CSF (Fig. 5B). The oxidative phosphorylation pathway showed increased activity in most cell types in PBMCs and CSF from the nonsurvivor, indicating metabolic changes associated with TBM mortality. The apoptosis gene set was enriched in several subsets of T cells, NK cells, and monocytes from the nonsurvivor, with highest activity in GNLY^+^CD8^+^ TEM, GZMK^+^CD8^+^ TEM, adaptive-like NK, CD4^+^ TM, MAIT, CD14^+^ monocytes, and CD16^+^ monocytes in PBMCs, and in highly inflammatory CD56^bright^ NK, GZMK^+^CD8^+^ TEM, activated GZMK^+^CD8^+^ TEM, GNLY^+^CD8^+^ TEM, and microglia-like macrophages in CSF (Fig. 5A, B).

## Discussion

Over the past decade, significant insights have been gained into the cellular and biochemical environment of CSF in CNS infection, particularly in TBM, contributing to advances in diagnosis and treatment. However, the complex biological processes and disease mechanisms remain poorly defined, necessitating high-resolution tools to capture the full immune landscape in the CSF. For the first time, we employed scRNA-seq in paired PBMCs and CSF from adults with TBM. We demonstrated the feasibility of using scRNA-seq in CSF, characterized the cellular composition in CSF, and uncovered distinct yet related cell and gene expression profiles in PBMCs and CSF in TBM.

We standardized protocols for studying CSF using scRNA-seq in multiple ways. First, we paired CSF with matched PBMCs from each patient, using the PBMCs as a reference. Second, we implemented a 2-step annotation process to confidently classify cell types. Third, we confirmed consistent major cell type proportions with flow cytometry. In total, we identified 15 major cell types and 29 cell subtypes in CSF, demonstrating a diversity comparable to that observed in PBMCs.

Single-cell transcriptional characterization of CSF from TBM patients showed differences with studies of other CNS diseases. In bacterial meningitis, neutrophils and monocytes were the main populations in the CSF.^14^ In contrast, CSF from patients with coronavirus disease 2019 was rich in T cells and CNS-associated macrophages.^35^ In multiple sclerosis, T cells made up the majority of cells in the CSF.^15^ In a previous study of children with TBM,^36^ CSF predominantly comprised 3 main populations, T cells (CD4^+^ and CD8^+^), B cells, and NK cells, consistent with our data. Thus, different cell types are mobilized to the CNS depending on the neuroinflammatory disorder.

In this study, we identified an abundance of *GZMK*-expressing CD8^+^ TEM and CD56^bright^ NK cells in the CSF compared with PBMCs. GZMK^+^CD8^+^ TEM are enriched in inflamed tissues, including CSF from children with TBM,^36^ pleural fluid from patients with tuberculous pleural effusion,^37^ and gut from patients with Crohn disease and ulcerative colitis.^38^ CD56^bright^ NK cells were the most abundant NK cell population in the CSF of TBM patients^39^ and in the CNS in multiple CNS disorders.^40^ While blood-abundant GNLY^+^CD8^+^ TEM, CD56^dim^ NK, and adaptive-like NK cells exhibited a classic cytotoxic phenotype in the CSF of TBM patients, GZMK^+^CD8^+^ TEM and CD56^bright^ NK cells showed low cytotoxic potential and enriched activity of cytokine-mediated signaling pathways. Such differences in the abundance of *GNLY*- and *GZMK*-expressing populations in PBMCs and CSF suggested a transition in cell phenotype driven by the local environment. Granzyme K is a serine protease released by cytotoxic lymphocytes that may induce apoptosis and contributes to the pathogenesis of inflammatory skin diseases, viral infections, and sepsis.^41–43^ Granzyme K itself could act as a key inflammatory factor by activating proinflammatory pathways, eg production of IL-6 and CCL2.^38^ Together, these findings suggest a crucial role for *GZMK*-expressing populations in driving inflammatory responses in the CSF. Future studies should explore the role of the *GZMK*-expressing cells, including GZMK^+^CD8^+^ TEM and CD56^bright^ NK cells, in TBM pathogenesis.

Along with *GZMK*-expressing cells, microglia-like macrophages, CD4^+^ TM, and Treg populations were increased in the CSF of TBM patients, providing new insights into TBM immunopathogenesis. Microglia-like macrophages in the CSF of TBM adults highly expressed genes for Toll-like receptors (*TLR2*/*4*/*7*), which are involved in *Mtb* recognition and phagocytosis, as well as complement-activating components (*C1QA*/*B*/*C*) and genes related to interferon (*CXCL9*/*10*) and inflammatory (*APOE*, *APOC1*) responses. The CSF of TBM patients was predominantly composed of specialized, tissue-resident and adaptive cell populations, while PBMCs showed relative enrichment of innate immune cell populations (eg monocytes) and naïve adaptive immune cells with high mobility. This distribution reflects the complementary and specific immunological roles of the 2 anatomical sites—blood cells circulate throughout the body and are prepared for rapid and systemic responses, whereas CSF cells are equipped to defend against pathogens on the frontline.

Compared with matched PBMCs, we observed increased inflammation and reduced oxidative phosphorylation in multiple cell types in CSF. Similarly, in a *Mycobacterium bovis*–induced TBM mouse model, scRNA-seq revealed decreased oxidative phosphorylation accompanied by increased activation of immune response and inflammatory pathways across diverse brain cell populations compared with uninfected mice.^44^ Our data also showed increased expression of *LDHA* (Table S3), suggesting enhanced conversion of pyruvate to lactate. This is consistent with previous reports indicating significant metabolic reprogramming in immune cells in TBM, characterized by a shift from oxidative phosphorylation to glycolysis under hypoxic conditions.^45^ A similar Warburg-like effect was observed in TB sputa relative to non-TB controls.^46^ This metabolic reprogramming may facilitate rapid ATP production to meet heightened energy demands during inflammation. Alternatively, it may reflect a functional shift in mitochondrial activity from ATP synthesis toward reactive oxygen species production as part of the host’s antimicrobial response.^46^ Together, these findings highlight the dynamic interplay between immune activation and energy metabolism across distinct tissue compartments in TB/TBM pathogenesis.

Compared with PBMCs from healthy donors, IFN-α and IFN-γ signaling pathways were elevated in both PBMCs and CSF from TBM patients, consistent with previous reports identifying IFN-driven responses as a hallmark of TB-associated immunity.^6^^,^^47^ These pathways were primarily enriched in subsets of T and NK cells across both compartments, suggesting that peripheral immune responses may partially reflect immune activity at the site of infection. Further investigation is warranted to elucidate the immunological interplay between blood and CSF in TBM.

Preliminary analysis of scRNA-seq data from the nonsurvivor compared to survivors revealed immune signatures consistent with previous bulk RNA-seq findings,^6^ including elevated inflammatory responses and TNF signaling in both PBMCs and CSF. Notably, the nonsurvivor was the only patient with a culture-confirmed *Mtb* infection, suggesting that these immune alterations may be linked to the presence of actively replicating bacteria—a known risk factor for immune dysregulation in HIV-associated TBM.^48^ scRNA-seq enabled the identification of specific immune cell populations potentially associated with poor outcomes. For instance, inflammatory signaling pathways were broadly upregulated across multiple cell types but not in B cells, CD4^+^ naïve/TCM cells, or CD8^+^ naïve/TCM cells. In contrast, the apoptosis pathway was specifically enriched in highly inflammatory subsets, including GZMK^+^CD8^+^ TEM cells, monocytes, and microglia-like macrophages, as well as in highly cytotoxic GNLY^+^CD8^+^ TEM cells, in PBMCs and CSF of the nonsurvivor. While these results require validation in larger cohorts, they highlight the potential of scRNA-seq to identify key cellular drivers of disease severity and mortality. Such insights may inform the development of prognostic biomarkers and targeted therapeutic strategies for TBM.

Our study has several limitations. First, the small sample size limited the statistical power to draw definitive conclusions. Nonetheless, it provided valuable proof-of-concept data supporting the feasibility of applying scRNA-seq to CSF samples in the context of TBM. Within the TBM cohort, only 2 patients had microbiologically confirmed disease. The prolonged turnaround time for culture results, coupled with the technical challenges of processing fresh CSF samples with low cell counts, prevented us from including only culture-positive cases. Additionally, only one patient in our cohort died, limiting our ability to investigate immune correlates of mortality. The absence of neutrophil-specific markers in our flow cytometry panel restricted our capacity to reconcile discrepancies between clinically assessed CSF neutrophil counts and those inferred from scRNA-seq data. Finally, our study did not include control groups with other CNS infections, such as viral or bacterial meningitis, which would be essential to determine the specificity of the immune signatures observed in TBM.

In conclusion, our study demonstrates the potential of scRNA-seq to explore compartment-specific immune responses in TBM. We successfully characterized the immune composition and gene expression profiles in PBMCs and CSF, revealing key differences between these compartments that may contribute to TBM mortality. The scRNA-seq workflow and analysis pipeline that we established here provides a valuable framework for future single-cell transcriptomic studies of blood and CSF to gain insights into the pathogenesis and identify potential treatment strategies for TBM and other brain infections.

## Supplementary Material

vkaf186_Supplementary_Data

## Data Availability

The sequencing data generated in this paper have been deposited in the National Center for Biotechnology Information’s Gene Expression Omnibus under the SuperSeries accession number GSE285118.
