# Supplementary material for: Single-cell profiling of blood and cerebrospinal fluid in tuberculous meningitis
Source: J Immunol. 2025 Aug 8;214(11):2894–905. doi: 10.1093/jimmun/vkaf186 (PMC12646059; doi:10.1093/jimmun/vkaf186)
Supplement: vkaf186_Supplementary_Data [file vkaf186_supplementary_data.zip › vkaf186_Supplementary_Data/vkaf186_Supplementary_Data.zip]

**Figure** **S1.** **Flow** **cytometry** **gating** **strategy**. Arrows depict the gating strategy. Mono: monocytes, CD4^+^: CD4^+^ T cells, CD8^+^: CD8^+^ T cells, T_Other: other CD3^+^ T cells, DCs: dendritic cells, cDCs: conventional dendritic cells, pDCs: plasmacytoid dendritic cells, NK: natural killer cells.

 **A**

**B**

**C**

**Figure** **S2.** **Quality** **control** **of** **single-cell** **RNA-sequencing** **data.** Violin plots of (A) the number of detected genes, (B) the number of detected mRNAs, or (C) the percentage of mitochondrial genes for each single cell from PBMCs and CSF from TBM patients. Low-quality or dead cells, defined as those with fewer than 500 detected genes and/or more than 10% of reads mapping to mitochondrial genes, were discarded before further analysis.

**Figure** **S3**. UMAP plot of Louvain clustering of combined PBMCs and CSF cells at a resolution of 1.9. Each cluster is color-coded and numbered.

**Figure** **S4.** Low number of detected genes and mRNA molecules in T_Other compared to the remaining T cell types. Lym_Prolif.: proliferative lymphocytes, TCM: T central memory cells, TEM: T effector memory cells, TM: T memory cells.

**Figure** **S5.** **Identification** **of** **cell** **subtypes** **(Level** **2)** **in** **PBMCs** **and** **CSF** **stratified** **by** **donor.** Cell subtypes were annotated based on expert knowledge. cDC1: conventional DC1, cDC2: conventional DC2, Gran: mixed granulocytes, Lym_Prolif.: proliferative lymphocytes, MegaK: megakaryocytes, MΦ: macrophages, Neutro: neutrophils, pDCs: plasmacytoid DCs, TCM: T central memory cells, TEM: T effector memory cells,TM: T memory cells, U/I: unidentified.

**Figure** **S6.** **Cell** **subtype** **proportions** **in** **PBMCs** **and** **CSF** **of** **individual** **patients.** Comparison of cell subtype proportions between PBMCs and CSF was performed using paired t-tests. *p < 0.05, **p < 0.01, ns: non-statistically significant difference. Adaptive_NK: adaptive-like NK, cDC1: conventional DC1, cDC2: conventional DC2, Gran: mixed granulocytes, Lym_Prolif: proliferative lymphocytes, MegaK: megakaryocytes, MΦ: macrophages, Neutro: neutrophils, pDCs: plasmacytoid DCs, TCM: T central memory cells, TEM: T effector memory cells, TM: T memory cells.

**A**	**B**

**C**

**Figure** **S7.** **k-NN** **weighted-network** **analysis** **to** **identify** **differentially** **abundant** **neighborhoods** **using** **miloR.** (**A**) UMAP plot of all cells colored by tissue of origin. (**B**) k-NN weighted-network analysis to identify differentially abundant neighborhoods using the miloR package. Nhood size indicates the number of cells in the neighborhood. (**C**) Volcano plot indicating differentially abundant (spatial FDR < 0.1) neighborhoods (n = 1845) between PBMCs and CSF.

**Figure** **S8.** **Gene** **expression** **levels** **in** **CD8^+^ T and** **NK** **subtypes.** Normalized expression levels of example differentially expressed genes between GZMK^+^CD8^+^ TEM and GNLY^+^CD8^+^ TEM cells (left), and between CD56bright NK and non-CD56bright NK cells (right).

**Figure** **S9.** **Identification** **of** **cell** **subtypes** **(Level** **2)** **in** **PBMCs** **from** **healthy** **donors** **and** **in** **PBMCs** **and** **CSF** **from** **TBM** **patients.** UMAP plot of high-resolution annotation (Level 2) of cell subtypes, based on expert knowledge for PBMCs from healthy donors (left), PBMCs from TBM patients (middle), or CSF from TBM patients (right).

cDC1: conventional DC1, cDC2: conventional DC2, Gran: mixed granulocytes, Lym_Prolif.: proliferative lymphocytes, MegaK: megakaryocytes, MΦ: macrophages, Neutro: neutrophils, pDCs: plasmacytoid dendritic cells, TCM: T central memory cells, TEM: T effector memory cells, TM: T memory cells, U/I: unidentified.
