# Supplementary material for: Single-cell profiling of blood and cerebrospinal fluid in tuberculous meningitis
Source: J Immunol. 2025 Aug 8;214(11):2894–905. doi: 10.1093/jimmun/vkaf186 (PMC12646059; doi:10.1093/jimmun/vkaf186)
Supplement: Supplementary file 1 [file vkaf186_supplementary_data.zip › vkaf186_Supplementary_Data/Supplementary_file_1_revised.odt]

Single-cell profiling of blood and cerebrospinal fluid in tuberculous meningitis

Supplementary File 1- Clinical descriptions of each patient suspected with tuberculosis meningitis (TBM)

Patient 1

A 40-year-old male, with a nine-day history of headache, vomiting and fever, followed by one day of altered consciousness. On hospital admission, his Glasgow Coma Scale (GCS) was 11. CSF appeared clear, with a white cell count of 382 cells/mm3, comprising 87% lymphocytes. CSF was positive for acid-fast bacilli on Ziehl-Neelsen (ZN) smear, and Mycobacterium tuberculosis (Mtb) was detected at trace level by Xpert MTB/RIF Ultra. Mycobacterial growth indicator tube (MGIT) culture, bacterial culture, India ink stain and cryptococcal lateral flow assay (LFA) were negative. The patient was diagnosed with modified British Medical Research Council (MRC) grade 2 TBM and achieved full recovery following standard anti-TB treatment.

Patient 2

A 60-year-old male was admitted to the hospital with a GCS of 13 and a three-week history of headache, along with two weeks of fever, altered consciousness and lethargy. He had a history of pleural TB treated 10 years previously, and no other significant medical history. CSF was positive for Mtb at the low level by Xpert MTB/RIF Ultra with no rifampin resistance detected and later confirmed positive by MGIT culture. CSF bacterial culture, India ink stain and LFA were all negative. He was diagnosed with MRC grade 2 TBM and died four days after initiating anti-TB treatment.

Patient 3

A 40-year-old male with a two-week history of headache and fever, was admitted to the hospital due to vomiting, urinary retention, and one day of altered consciousness. CSF appeared clear, with a white cell count of 330 cells/mm3, comprising 100% lymphocytes. CSF ZN smear, Xpert MTB/RIF Ultra, MGIT culture, bacterial culture, India ink stain and LFA were all negative. CSF analysis revealed elevated protein (1.45 g/L), reduced glucose (4 mmol/L in CSF vs 9.1 mmol/L in serum). The prolonged clinical course for two weeks and CSF characteristics were suggestive of MRC grade 1 TBM. He responded well to anti-TB treatment and achieved full recovery by 12 months.

Patient 4

A 53-year-old male, with a two-week history of headache and fever, presented to the hospital due to neck stiffness. On admission, his GCS was 14. CSF appeared clear, with a white cell count of 357 cells/mm3, comprising 92% lymphocytes. CSF analysis revealed elevated protein (1.01 g/L), reduced glucose (2.17 mmol/L in CSF vs 5.8 mmol/L in serum). CSF ZN smear, Xpert MTB/RIF Ultra, bacterial culture, India ink stain and LFA were all negative. Chest X-ray suggested pulmonary TB, however sputum ZN smear and Xpert MTB/RIF Ultra were negative. He was diagnosed with grade 2 TBM, smear negative PTB, and achieved full recovery following standard anti-TB treatment.
